# Advancing the Science of Recruitment for Family Caregivers: Focus Group and Delphi Methods

**DOI:** 10.2196/13862

**Published:** 2019-07-22

**Authors:** Dana Hansen, Amy Petrinec, Mona Hebeshy, Denice Sheehan, Barbara L Drew

**Affiliations:** 1 College of Nursing Kent State University Kent, OH United States; 2 Faculty of Nursing Suez Canal University Ismailia Egypt

**Keywords:** family caregivers, recruitment, social media, illness stories

## Abstract

**Background:**

Successful recruitment of participants is imperative to a rigorous study, and recruitment challenges are not new to researchers. Many researchers have used social media successfully to recruit study participants. However, challenges remain for effective online social media recruitment for some populations.

**Objective:**

Using a multistep approach that included a focus group and Delphi method, researchers performed this study to gain expert advice regarding material development for social media recruitment and to test the recruitment material with the target population.

**Methods:**

In the first phase, we conducted a focus group with 5 social media experts to identify critical elements for effective social media recruitment material. Utilizing the Delphi method with 5 family caregivers, we conducted the second phase to reach consensus regarding effective recruitment videos.

**Results:**

Phase I utilized a focus group that resulted in identification of three barriers related to social media recruitment, including lack of staff and resources, issues with restrictive algorithms, and not standing out in the crowd. Phase II used the Delphi method. At the completion of Delphi Round 1, 5 Delphi participants received a summary of the analysis for feedback and agreement with our summary. Using data and recommendations from Round 1, researchers created two new recruitment videos with additions to improve trustworthiness and transparency, such as the university’s logo. In Round 2 of the Delphi method, consensus regarding the quality and trustworthiness of the recruitment videos reached 100%.

**Conclusions:**

One of the primary challenges for family caregiver research is recruitment. Despite the broad adoption of social media marketing approaches, the effectiveness of online recruitment strategies needs further investigation.

## Introduction

### Overview

Social media use is widespread across generations, with 68% of Americans using Facebook and 73% accessing YouTube. Twitter is gaining popularity with adult Americans (14%) compared with already-engaged younger adults (45%) utilizing this social media platform [1]. Social media promotes communication, interaction, collaboration, and sharing [2]. The flexibility of communication inherent to social media platforms has led to their increased use in health and health care, including family caregivers caring for ill loved ones [3,4].

The social media illness story is a modern form of interaction that encourages relationship development and encapsulates a rich narrative that holds untapped resources for understanding the psychological, physical, spiritual, and social impact of the patient’s illness journey [5]. Some ill people write an illness blog using a blog platform; others use Facebook or a microblog format like Twitter to tell their illness stories. This form of online journaling has been found to be beneficial for patients and their family caregivers [3,6,7]. For example, online journaling is known to bring meaning of the illness to both the patient and family caregiver. Social media is also becoming a successful and popular venue for recruitment [2,5]. However, in previous research, the authors found recruiting family caregivers whose loved ones tell their illness stories on social media challenging since this small subset of family caregivers is difficult to define and identify. Although family caregivers are included in many illness stories on social media, they are not easy to identify.

Understanding and contributing to the science of social media recruitment for family caregivers with a loved one who tells their illness story on social media is paramount to advancing family caregiver recruitment. To explore challenges to successful recruitment of family caregivers through social media, we used two companion approaches. In the first phase of the study, we conducted a focus group with social media experts to identify critical elements of effective social media recruitment material. Utilizing the Delphi method, the second phase of the study confirmed that the product created aligned with focus group feedback.

### Background

Successful recruitment of participants is imperative to a rigorous study, but recruitment challenges are not new to researchers. In fact, failed recruitment efforts result in underpowered studies and nonsignificant findings [8]. Theoretical development surrounding response behavior in research found that poor response rates were associated with socioeconomic demographics for underrepresented participants, such as women [9]. However, efforts to evaluate study participation recruitment methods through varied questionnaire distribution choices, such as Internet distribution, have shown promise [10].

Many researchers have successfully recruited study participants through social media, such as a Facebook advertisement feature that utilizes a targeted format (eg, researchers select specific demographics and keywords) [11]. Even researchers with targeted and small populations [8], such as our study population of family caregivers with loved ones who tell their illness stories on social media, have had success with social media recruitment [12-14]. Furthermore, several studies have concluded that online recruitment is more cost-effective than traditional strategies [15-17]. However, challenges remain with recruitment for family caregivers who read or interact with their seriously ill loved ones online through patient illness stories.

### Previous Work

In our previous work, we found that a patient’s illness blog assisted the family caregiver with communication, creation of meaning, and identification of their role as caregiver [3]; however, we encountered several recruitment challenges for this population when using a Facebook campaign [11]. While serious illness affects many Americans, issues confronted by family caregivers during serious illness comprise a sensitive topic that may not be of interest to social media users. Therefore, we did not obtain a high-enough click-through rate by potential study participants to maintain a successful Facebook recruitment campaign [11]. Additionally, the population recruited was a narrow subgroup of family caregivers (ie, those who read their loved one’s illness story online) who were difficult to locate. Finally, technical skills involved with social media recruitment are not typically taught to nurse researchers, which means collaboration outside of nursing is required.

In this study, we sought to develop our understanding of the science of recruitment of family caregivers through social media. Using a data-based, multistep approach, we gained a more complete understanding of the science of recruitment for our population, improving the likelihood of reaching our target population and ensuring their engagement. The purpose for the focus group was to gain meaningful insight, opinions, suggestions, and feedback to develop social media-based recruitment methods for future research studies. For the Delphi method, the purpose was to test the developed material to gather insights, opinions, and suggestions from our target population: family caregivers of people who tell their illness stories on social media.

## Methods

### Overview

Institutional Review Board approval was obtained through Kent State University, Kent, OH, prior to conducting all aspects of the study (approval number: 16-226). All participants were provided with project information and gave consent to participate in this two-phase study. In the focus group (Phase I), social media experts provided expert opinions for both development of social media recruitment materials and barriers to recruiting family caregivers with loved ones who tell their illness stories on social media [18]. In Phase II, the Delphi method containing quantitative and open-ended questions was used to obtain feedback on the quality, trustworthiness, and clarity of the newly developed recruitment material [19,20].

### Focus Group: Phase I

#### Sampling

Researchers used purposive sampling methods to recruit 5 social media experts through email messages sent to department heads of a major Northeast Ohio public university, including Journalism and Mass Communications, Web and Social Media Services, the Marketing Department of the College of Business, and other departments with researchers engaged with social media in research. Email messages explained the purpose of the focus group and time commitment, with a request to nominate potential faculty members that met the following inclusion criteria: (1) expertise in social media (ie, able to contact targeted populations using social media recruitment) and (2) representation by both male and female members.

The 5 peer-nominated expert faculty—3 female (60%) and 2 male (40%) participants—comprising the focus group have a record of accomplishment in social media, including capturing and analyzing social data metrics. The group’s expertise included a faculty member from the Alumni Association Department with knowledge of online marketing and engagement strategies, a researcher from the Department of Sociology with experience recruiting men with gynecomastia, two faculty members in the Public Relations Department taught social media usage, and one faculty member contributed expertise in education and instructional design.

#### Setting

The focus group ran for 2 hours in a seminar room at the university. To engage participants in a natural conversation, we began the focus group with introductions, presented Facebook advertisement material used in our prior research, and described the purpose of our research and need for effective recruitment materials.

#### Interview

Serving as moderator, one of the experienced qualitative researchers (BLD) initiated the audio-recorded, semistructured discussion by asking specific questions about methods used for our previous study (eg, “What do you think about those methods and do you have suggestions for improvement?”). We also asked broad questions about social media (eg, “Tell us about your experiences with social media and social media recruitment.”) and facilitated interaction among the group with prompts and redirection. Specific questions about social media recruitment dealt with characteristics that make for high-quality social media recruitment materials, best options for recruitment strategies, and barriers to effective social media recruitment.

#### Analysis

Researchers conducted focus group analysis following descriptive qualitative methods [21], and the principal investigator (DH) verified and transcribed the recorded focus group session verbatim. Four researchers from the university’s College of Nursing, two with expertise in focus groups and Delphi methods and two with expertise in caregiver research, individually analyzed the focus group data utilizing descriptive qualitative methods. Each researcher categorized the themes, and researchers met twice to discuss the findings. Researchers modified the thematic structure throughout the analysis until reaching consensus [22]. To ensure trustworthiness, we conducted a member check; the findings were sent to each focus group participant for verification, and each focus group participant agreed that the analysis represented his or her views. Therefore, final themes represent the authentic view of the participants’ discussion.

### Delphi Method: Phase II

#### Overview

Using the online Delphi method, we accessed expert stakeholders—family caregivers with seriously ill loved ones who tell their illness stories on social media—from a large geographic area for differing perspectives. The Delphi method—a group facilitation technique featuring an iterative, multistage process—is frequently used to reach consensus. The Delphi method allows for multiple rounds of questions to a group of experts, providing an opportunity to improve the product, in this case recruitment videos, while building consensus among participants regarding important characteristics of the recruitment material. Although there is no universal agreement for what constitutes consensus, the typical range of percent agreement (ie, the standard for Delphi methods) is from 51% to 80% or more [23].

#### Sampling

The Delphi technique utilizes purposive sampling to identify experts, defined as informed individuals specializing in the field or who have knowledge about the subject [24]. For the Delphi phase of this study, experts were identified as family caregivers with loved ones who use social media to tell their illness stories. This subset of family caregivers was narrowly defined and therefore difficult to recruit. In previous research, we found that women tended to tell their illness stories publicly more often than men, making men the more common caregivers for our study sample, although not typical of the general caregiver population [3,11]. Therefore, our study sample is representative of this subset of family caregivers whose loved ones tell their illness stories on social media. There is no universal agreement on the proper size of the expert panel for Delphi methods; however, panels have ranged in size from 4 to more than 3000 participants [25,26]. Regardless of the large range of sample sizes in research studies, 5-10 experts are suggested [26].

Family caregivers from our previous studies who elected to be contacted regarding future research opportunities were asked if they would like to participate in the Delphi portion of our study. A total of 3 out of 9 family caregivers consented to participate. In addition, 2 more participants were recruited through snowball sampling, a nonprobability sampling technique in which an existing participant recruits other participants because of their acquaintance, resulting in 5 family caregiver participants. All 5 participants were 18 years old or older, with loved ones with a serious illness who use social media to communicate about their illness. Trustworthiness was established with a member check of our analysis of each round [27]. Researchers conducted a member check by sending a summary of the results from each round and asking family caregivers to confirm the summary or add any necessary clarifications.

#### Qualtrics Survey

The Round I Qualtrics survey began with a consent form explaining the study; those who agreed to participate were taken to the online survey. The first part of the survey requested demographic information and then introduced six open-ended questions: (1) What kind of recruitment practices interest you the most?; (2) What drew your interest to participate in this research study?; (3) What is important in recruiting family caregivers to participate in a research study?; (4) In your opinion, what qualities make you trust an online advertisement?; (5) What recommendations do you have for improving study recruitment materials?; and (6) What social media sites do you visit most frequently? In other words, to which social media sites should we post recruitment videos?

To ascertain what motivates family caregivers to participate in research studies, we asked respondents to rate the following statements on a 5-point Likert scale, from 1 (not at all agree) to 5 (strongly agree):

1. What is the likelihood of participating in a research study that uses online recruitment strategies?
2. I participated in this research study because I believe in advancing science through participation in studies.
3. I participated in this research study because I received compensation.

Participants were then asked to watch recruitment video 1 and answer questions about video quality, trustworthiness, whether the viewer was encouraged to visit the website, and whether the viewer would answer the call to action by clicking on the landing page (ie, the website that explains the research). Participants were asked the same questions about video 2. The final question of the survey asked about the time of day participants typically use social media and are therefore more likely to see recruitment ads.

Following Round 1, researchers sent a summary of Round 1 analysis to the 5 Delphi participants, who then provided feedback as to whether they agreed with our summary and were given the opportunity to offer clarification. Using data and recommendations from Round 1, we sought the assistance of a professional graphic designer to develop two new recruitment videos related to recruiting family caregivers to participate in research. Following completion of the videos, we conducted Round 2 of the Delphi method by sending a new Qualtrics survey with links to the new recruitment videos to Delphi participants and encouraged them to provide feedback for those videos.

#### Validity and Delphi Analysis

Content validity in Delphi methods is achieved utilizing expert panel members, and successive rounds increase concurrent validity [23]. To ensure the accuracy of Delphi results, we confirmed that the questions and instructions were clear. After Round I, we discovered that participants may have misunderstood one of the questions, so we reworded the question and provided further clarification to ensure understanding. We followed up with one nonrespondent, accurately coded the survey data, recorded all qualitative and quantitative data, and verified the data with a member check after each round.

The same four researchers who analyzed focus group data individually analyzed quantitative and qualitative data from Round I of the Delphi method. A research assistant generated a report from Qualtrics yielding descriptive data for quantitative questions and created a Word document listing the open-ended questions and answers from all participants.

## Results

### Focus Group: Phase I

#### Overview

Opening discussion with focus group panelists was rich and offered opportunities to clarify the purpose of the research and the intended audience for recruitment material. Nonetheless, panelists did express some confusion regarding the study. For example, one participant asked,

What is the relationship between the caregiver and their experiences as a caregiver and then the loved one’s blog?

Another participant inquired,

So we’re focusing primarily on the person that’s sick who’s blogging, not necessarily the family caregiver that’s blogging?

Researchers clarified that although patients are telling their stories, recruitment centers on family caregivers of those patients who are interacting with or reading their loved one’s blog. Another participant acknowledged the difficulty inherent to recruiting this subset of family caregivers, acknowledging,

So, that’s what makes this complicated; you are depending on the ill person to relay the information to the caregiver and it’s a very focused target group. So, that’s the challenge.

The results of the analysis fell into three overarching categories: barriers, effective social media material, and need for a landing page.

#### Barriers

During analysis of focus group data, three barriers to effective family caregiver recruitment were identified, including lack of staff and resources, issues with restrictive algorithms, and not standing out in the crowd.

##### Lack of Staff and Resources

Lack of staff and resources was identified as a barrier due to the time required to manage the social media accounts. Without infrastructure to support resources and the necessary research staff, in terms of skill level, it is difficult to maintain active social media accounts that include frequent postings and identification of appropriate algorithms for recruitment. This scarcity was supported by the focus group participant comment,

The resources are barriers in terms of staff time and folks that are nurturing these accounts.

Another participant stated that an inactive social media account is a “red flag” to potential participants, adding, “...ideally, the account is active [has postings] one-to-three times per week.” One participant expressed his concerns regarding inactive accounts, asking,

So is it [the Facebook page used in previous research] inactive? Because that raises a red flag for me if this page is only running advertisements and you might post once every three months—that’s technically like an inactive page and your user does not want to see that.

Another resource identified as a barrier was the budget. A participant with a background in recruiting alumni to events stated,

Budget—that was a big one and then actually converting clicks into action...We get a lot of clicks on our ads, but if we have 300 clicks on one of our ads, we might get one or two RSVPs from that. So, the ads are usually good because they’re working, they’re getting lots of clicks, and the cost per click is low, but then how do we get them to RSVP to an event, take the action that we want them to do.

Another participant noted,

So, that’s even why it’s more important to pay to play with these [ads] because you can gradually get right in front of people. So, the fact that you guys are already started with advertising is a good strategy.

##### Algorithms

Algorithms were identified as barriers to family caregiver recruitment because the researcher is limited to allowable criteria determined by the social media venue. For example, on Facebook, one can target a recruitment advertisement to a certain age or even certain words, such as cancer or illness. Since we are targeting a very specific group of family caregivers (ie, those who have loved ones who tell their illness stories on social media), it was difficult to find accurate identifiers. This complexity was voiced by one participant who stated,

There’s a fine line because you don’t want to post too much on social media because then you start to turn people away and they’ll tune you out. Targeting is a huge barrier, like you said; to get down to exactly who you need to be in front of takes a lot of fine tuning—there’s age, demographics, interest, there’s all that.

Another participant noted, “Another barrier is the algorithms because that dictates who sees your content.”

##### Not Standing Out in the Crowd

Focus group participants identified not standing out in the crowd as another potential barrier to successful recruitment. Despite the necessity of drawing potential study participants to the social media account, focus group participants recognized that the topic of death and dying might not draw a large audience. One participant explained,

We approach it more from the perspective of the person in terms of why this would be valuable to them to participate. For example, you’ll have the opportunity to share your voice and how this impacts your life or something along those lines because I’m sure that they are impacted by this.

However, having a unique message helps to be recognized and stand out in a crowd. Another participant noted that,

The content of the ad is a really critical part of the social media strategy in terms of creating content that’s valuable, and if you can showcase the value here [in the recruitment ad], then that’s when it brings others, too.

Another participant validated this comment by stating,

Something else that I noticed [about recruitment material previously used] was all of these headlines say we want to interview you. Whereas this headline [the one that drew the most attention] was ‘Is your loved one seriously ill?’ I think that’s a better catch.

#### Effective Social Media Material

Creating effective social media material was another theme discussed by participants. Three supporting subthemes for effective social media material included the need to connect on a personal level with the target audience, the need to use real photos instead of stock photos, and the need to create a crystal-clear message. Focus group participants strongly stressed the need for a personal connection with potential study participants to encourage more robust online recruitment. One focus group member stated that the audience must believe researchers are addressing them individually, suggesting that researchers ask questions that elicit the response, “Yeah, that’s me, yeah that’s me, yeah that’s me. We want you to click here!” According to the focus group, another critical part of the social media strategy in terms of creating content is to “showcase the value—that is what brings others to it [the social media advertisement].” One participant with experience in public relations stated,

Provide value and what’s in it for you, that’s the mantra in public relations. So, I wonder if we approach it more from the perspective of the person in terms of why this would be valuable to them to participate in the study versus we want to interview you, maybe you’ll have the opportunity to share your voice and how this impacts your life or something along those lines because I’m sure that they are impacted by this [caregiving experience].

Another focus group participant noted,

You pointed out defining a caregiver, I think that’s important so, if there would be a way to work that in somehow, I think that would be good.

Focus group participants also identified the importance of using real photos instead of stock photos. Real photos elicit a more personal connection, and stock photos are considered a red flag to the potential participants. One focus group member noted that,

When they [potential study participants] see stock images—they [potential study participants] know that’s an ad. So, they’ll scroll right past it...I think that you need more compelling images that are going to catch somebody’s attention.

Participants expressed the need for a crystal-clear message to engage the audience with a call to action. The call to action motivates the audience to click on the link that takes them to the study’s landing page. Potential study participants must feel compelled to click on the link. One method identified by focus group participants to deliver a clear message containing a call to action is through a video message. Video messages do not have word limits as do Facebook advertisements, allowing researchers to explain the research in more depth and create a more personal message. Focus group participants offered various strategies for using video messaging to improve family caregiver recruitment, including the following guidance:

...take your iPhone, record something and post it in these forums and then steer them to the landing page.

Facebook is really rewarding people who use video advertisements right now and you get more bang for your buck.

Use organic posting for Facebook and Twitter, not just advertisements—if you create a 30-second video, you can upload that directly to Twitter and you will see a lot of engagement because it autoplays just like it would on Facebook.

#### Landing Page

Finally, focus group participants suggested creating a study landing page for potential research participants that is user friendly, expands on the clear message, and presents a call to action as a way to connect all recruitment material. One participant highlighted the need for “making it simple and making that call to action very clear so it takes them to the landing page.” Another participant agreed with the necessity of a study landing page, stating,

...once you get them to that page where they are actually converting, that has to be simple, too. So, that landing page is another critical piece.

Participants also suggested creating a hashtag that goes out with every recruitment effort to connect the recruitment material, explaining that “The hashtag will take them back to the landing page.” This landing page can be an active social media account, such as Facebook, or an active webpage.

After compiling focus group recommendations, work began to create new social media recruitment material. We decided to create recruitment videos that would take potential participants to our study landing page, which provides information about current studies and how to become part of our caregiver registry. The principal investigator created two videos using a program called Biteable [28]. These two videos included no audible words but displayed pictures of people representing the target population. Words describing the purpose of the video and how to contact the researcher were displayed on the screen, and music played in the background.

### Delphi Method: Phase II

#### Overview

Using the Delphi method, the updated recruitment videos were tested with a sample of 5 family caregivers who have a loved one who tells their illness story on social media. Delphi participants ranged in age from 57 to 70 years with a mean age of 58 (SD 7). Caregivers were predominantly male (4/5, 80%) with an average of 3 years of caregiving experience (see Table 1). Participants’ responses to reasons for participating in the study and the importance of recruiting family caregivers in research were varied (see Textbox 1).

**Table 1:** Demographics of participants for the Delphi method.

Demographics		Value
Age (years), mean (SD)		58 (7)
**Gender (n=5), n (%)**		
	Male	4 (80)
	Female	1 (20)
Years of caregiving, mean^a^		3

^a^SD is not provided for years of caregiving because responses were ranked options (eg, 0-2 or 3-5 years).

**Participants’ open-ended responses from round 1 of the Delphi method.**

Participants’ reasons for participating in this study:

- Participants’ awareness of positive results that come from research
- Son uses Facebook and Caring Bridge to talk about his illness journey
- Was asked by a friend
- Experience as caregiver
- Happy to contribute our experiences to others

Importance of recruiting family caregivers to participate in research:

- So that caregivers know what kind of help is available and to help others
- We would not be where we are today in the medical field if studies were not conducted
- Explaining the goals of the research
- Caregivers are frontline workers and need support, including emotional support
- Diversity—it’s best when a study covers a wide range of circumstances

#### Delphi Round 1

For each video, Delphi participants were asked to rate specific characteristics, including the overall quality, trustworthiness, the extent to which the video encourages or discourages participants to seek more information, and, finally, the likelihood that participants would act on the video’s request (ie, to “click here”). Participants rated each characteristic as *poor*, *fair*, *medium*, *high*, or *highest*.

The first round of videos yielded encouraging results that offered opportunities for improvement. Both videos did not have lower than *medium* rating scores, although the overall quality was slightly higher in video 1. Video 2 had a slightly higher score for trustworthiness compared to video 1, and both videos had one rating of *poor* for trustworthiness. Neither video was ranked well in encouraging participants to seek more information; however, video 2 performed worse than video 1 in this category. For both videos, more participants said that they would not “click here.”

We also asked several open-ended questions. In terms of when family caregiver participants would be most likely to see a recruitment video on social media, 3 out of 5 participants (60%) reported 5:00-8:00 am as the most likely time, another participant (1/5, 20%) chose 1:00-3:00 pm, and one other participant (1/5, 20%) was more likely to see a recruitment video from 8:00-10:00 pm. Participants identified newspaper ads, social media posts, fliers in physicians’ offices and waiting rooms, and email messages as the most appealing recruitment practices.

In their responses to the question, “What is important in recruiting family caregivers to participate in a research study?” participants identified three factors that facilitate trust for recruitment videos on social media. These factors focused on the family caregiver’s ability to trust the organization and for the organization to do the following: (1) ensure the source of the recruitment material is clear and visible on the recruitment material, which includes a recognizable logo; (2) improve the clarity of the research purpose presented on the recruitment material; and (3) use known social media sites such as Facebook, Twitter, and Instagram. Participants stated that they participate in research studies because they believed in advancing science through research and suggested posting on disease-specific sites, such as the National Multiple Sclerosis Society website or the Stem Cell Transplant Group Facebook page.

Prior to moving forward with Round 2, we consulted with a researcher with expertise in recruiting participants at the end-of-life stage and conducting research with social media illness stories. She reviewed the videos from Round 1 and the newly developed Round 2 videos, along with the scripts for each video. Her feedback was incorporated into the final production of Round 2 videos.

#### Delphi Round 2

In Round 2, participants were asked the same questions about characteristics of the videos to allow us to analyze issues identified with the videos in Round 1. Ratings improved to above a *medium* score for each video in all characteristics, with video 2 achieving slightly higher overall ratings.

Overall, recommendations provided to improve Round 2 recruitment videos were successful, as 100% consensus was achieved. For example, one participant commented, “These ads were a big improvement, very clear and to the point.” We summarized Round 2 feedback and sent that summary to Delphi participants, who confirmed consensus. Comments from this group included the following statements:

All good points on the feedback.

Participant #1

With the new videos and new feedback, your videos should work well.

Participant #1

Thank you for letting me participate.

Participant #1

I would agree with the summation.

Participant #2

Good summary and you did capture my feedback.

Participant #3

I think you have correctly captured my comments; thank you.

Participant #4

Some suggestions mentioned the addition of closed captioning, which has been completed.

## Discussion

### Principal Findings

Family caregivers at the end-of-life stage provide important support for the dying patient; they also need vital support to better help their loved ones. However, recruitment for this population is complex, especially for a subset of family caregivers whose loved ones tell their illness stories on social media. This study offers insights on recruitment for this subset of caregivers. Focus group participants and Delphi participants validated that social media is an important venue for participant recruitment and offered critical suggestions for effective social media recruitment material for family caregivers. These recommendations include identification of barriers, creation of effective recruitment material, and the need for a landing page prior to recruitment efforts. Development of effective recruitment material includes ensuring the identity of the organization, improving trustworthiness and transparency, and ensuring that the purpose of recruitment is clear.

Social media algorithms determine which advertisements align with users’ interests based on their onsite activity, listed interests, and interactions. Creating a video with a focused and targeted audience is required to take full advantage of social media advertising. For example, using real photos instead of stock images builds a connection with the target audience, which leads to greater trust [29]. Our findings revealed that algorithms were identified as barriers to social media recruitment for our study population of interest, as researchers are limited to criteria of interest for a specific group of family caregivers (ie, those who have loved ones who tell their illness stories on social media). These findings remain consistent with a systematic review reporting similar limitations in other study populations, because using ads on social media websites requires targeting specific age groups and locations based only on the information an individual provides on his or her profile [30]. Therefore, there is no guarantee that awareness of the study reached all potential participants, which introduces bias into the results.

Focus group participants acknowledged that maintaining active accounts and updated valuable ad content are critical components of social media to draw a large audience. Our findings validate Pang and colleagues’ findings that unless researchers utilize transparent and relevant information for health care consumers, online and social media platforms used in recruitment will not command traffic [29]. In our study, we found an emphasis on the need to make a personal connection with potential study participants so they may receive the “call for action” and the necessity for a clear message to engage participants and maintain account activity. Our focus group participants (Phase I) discussed the importance of delivering a clear message so that potential participants could easily identify with the research. Akers and Gordon also emphasized the importance of linking the study recruitment advertisement to the study URL (ie, the place where participants are directed when they click on the ad containing details of the study) and the Facebook landing page [31].

Howcutt and colleagues propose a marketing framework to improve recruitment [32]. Combined perspectives of marketing science and behavioral science focus on persuasion and decision making. Success with marketing employs strategies to connect the researcher to the participant, understanding that both receive a benefit from the relationship. However, successful recruitment occurs when there is an emphasis on the “consumer” or on the potential participant’s needs, decreasing barriers to research participation and improving participant motivation to engage [32,33]. In the Delphi phase, we found that the caregivers were not motivated by compensation but, rather, were motivated by the idea of making a difference for others. Similar to our findings, it is suggested that emphasis be placed on the commonalities of the population (eg, caregivers’ desire to share experiences) instead of manipulating behavior to fulfill researcher goals [33].

Howcutt’s framework includes attending to perceptions and attitudes of participants, which may influence family caregiver recruitment [32]. As an example, some people base decisions on consideration of the facts and reflection on the benefits and burdens of participation. We know that our target population of family caregivers does not have an abundance of free time. However, perceptions and attitudes affect how individuals consider participation in an activity, which may include identifying time constraints of caregiving as burdensome, thus increasing that burden. However, if caregivers perceive the benefit of research participation to help others as more important than time constraints, caregivers may be more likely to engage in research. Family caregivers who participated in the Delphi method perceived research participation as important to helping others. Therefore, another layer of complexity is added when researchers must also consider the variety of perceptions and attitudes held by their target population.

Integration is a concept in Howcutt and colleagues’ discussion that is likened to what our participants labeled a “call to action.” The call to action is influenced by what Ajzen [34], in his seminal work, referred to as a person’s intent to adopt a new behavior. By understanding and removing barriers as uncovered in our focus group of social media experts, we can turn interest or intent by potential study participants into action.

In addition to extending a new perspective to examine the importance of social media relationships, social network sites offer researchers from a variety of disciplines a unique venue for recruitment [35]. Challenges with participant recruitment are often the primary reason for study delays [36]. Using social media for recruitment improves the capacity of sampling while minimizing the cost of obtaining large sample sizes, thereby increasing access to hard-to-reach populations, such as caregivers of patients with serious illness.

### Limitations

While the purposive sampling method allowed for participation of peer-nominated participants with expertise in social media recruitment and advertisement as well as expert stakeholders in the Delphi method, the findings are not generalizable. In addition, the focus group method presents challenges associated with collecting data. These challenges may arise from the nature of questions posed by the moderator, or a more gregarious participant may drive the direction of the discussion. However, the member check allowed us to assess if these issues existed in our study. All participants agreed with our data analysis and did not offer additional insight.

With Delphi methods, there is no evidence of reliability. For example, we do not know whether study results would be the same if identical information was given to another panel of family caregivers. However, validity is also an issue when using the Delphi method and arises from pressures put on panel members to change their opinions according to the group response [23]. Our participants did not change their responses after the summary was provided; therefore, we are confident in the validity of our findings in this sample. Typically, there are at least three rounds in a Delphi method to reach consensus. However, we believe we were able to reach consensus after two rounds because of the initial focus group data that was used to develop recruitment material presented to Delphi participants.

### Conclusions

One of the primary challenges to conducting research with family caregivers is recruitment. Recruitment through social media is a promising means of engaging family caregivers in research and may be a cost-effective alternative in recruiting hard-to-reach populations [36]. Furthermore, because of widespread use of social media, there are fewer geographical boundaries for Internet recruiting, which may improve generalizability of research studies. This study contributes a unique view of the science for building effective videos to recruit family caregivers. Videos offer a short and clear message about the research, and the visual aid assists consumers with learning and understanding the content [29]. The initial focus group comprised of social media experts helped us develop targeted recruitment materials to overcome barriers to social media recruitment with family caregivers. The Delphi portion of this study allowed us to gain valuable feedback on the new recruitment material and then adapt that material based on participant feedback.

Despite the broad adoption of social media marketing approaches, the effectiveness of different online recruitment strategies needs further investigation. Future research should focus on the utility of various social media sites for recruitment purposes
